# In Utero Alcohol and Unsuitable Home Environmental Exposure Combined with *FMR1* Full Mutation Allele Cause Severe Fragile X Syndrome Phenotypes

**DOI:** 10.3390/ijms26072840

**Published:** 2025-03-21

**Authors:** Tri Indah Winarni, Ramkumar Aishworiya, Hannah Culpepper, Marwa Zafarullah, Guadalupe Mendoza, Tanaporn Jasmine Wilaisakditipakorn, Narueporn Likhitweerawong, Julie Law, Randi Hagerman, Flora Tassone

**Affiliations:** 1 Center for Biomedical Research (CEBIOR), Faculty of Medicine, Universitas Diponegoro, Semarang 50275, Central Java, Indonesia; triindahw@gmail.com; 2 Khoo Teck Puat-National University Children’s Medical Institute, National University Health System, Singapore 119074, Singapore; paearam@nus.edu.sg; 3 Department of Pediatrics, Yong Loo Lin School of Medicine, National University of Singapore, Singapore 117597, Singapore; 4 Department of Biochemistry and Molecular Medicine, University of California Davis School of Medicine, Sacramento, CA 95817, USA; hculpepper@ucdavis.edu (H.C.); mzafarullah@ucdavis.edu (M.Z.); gmendozamorales@ucdavis.edu (G.M.); 5 Department of Pediatrics, University of California Davis School of Medicine, Sacramento, CA 95817, USA; tjwilaisak@ucdavis.edu (T.J.W.); jmlaw@ucdavis.edu (J.L.); rjhagerman@ucdavis.edu (R.H.); 6 MIND Institute, University of California Davis Medical Center, Sacramento, CA 95817, USA; nlikhitweerawong@ucdavis.edu; 7 Department of Pediatrics, Faculty of Medicine, Chiang Mai University, Chiang Mai 50200, Thailand

**Keywords:** autism spectrum disorder, CGG repeat expansion, mosaicism, fetal alcohol spectrum disorder, fragile X syndrome

## Abstract

We investigated the molecular and clinical profile of five boys carrying the fragile X messenger ribonucleoprotein 1 (*FMR1*) mutation and who suffered from the effects of prenatal alcohol exposure. Fragile X syndrome (FXS) testing was performed using PCR and Southern Blot analysis, and fragile X messenger ribonucleoprotein protein (FMRP) expression levels were measured by Western blot analysis. Clinical evaluation included cognitive functions, adaptive skills, autism phenotype, and severity of behavior measures. Fetal Alcohol Spectrum Disorder (FASD) was also assessed. Five adopted male siblings were investigated, four of which (cases 1, 2, 3, and 4) were diagnosed with FXS, FASD, and ASD, and one, the fraternal triplet (case 5), was diagnosed with FASD and ASD and no FXS. The molecular profile of case 1 and 2 showed the presence of a hypermethylated full mutation (FM) and the resulting absence of FMRP. Cases 3 and 4 (identical twins) were FM-size mosaics (for the presence of an FM and a deleted allele), resulting in 16% and 50% FMRP expression levels, respectively. FMRP expression level was normal in case 5 (fraternal twin). Severe behavioral problems were observed in all cases, including aggression, tantrum, self-harming, anxiety, and defiant behavior, due to different mutations of the *FMR1* gene, in addition to biological exposure, home environmental factors, and potentially to additional background gene effects.

## 1. Introduction

Fragile X syndrome (FXS) is the most common cause of inherited intellectual disability (ID) and a single genetic cause of autism spectrum disorder (ASD) which is observed in 60% of males and 20% of females with FXS [1]. Affecting approximately 1: 2500 males and 1: 8000 females [2], it is mostly caused by the loss of the encoded product (FMRP) of the fragile X messenger ribonucleoprotein 1 (*FMR1*) gene located at Xq27.3. The presence of an allele harboring a CGG repeat number greater than 200, called a full mutation (FM), leads to hypermethylation and the inhibition of the transcription of the fragile X messenger ribonucleic acid 1 gene (*FMR1*), resulting in the absence of FMRP [3]

The lack of FMRP, an mRNA-binding protein with functions in mRNA transport, localization, and translational control in synapses, leads to dysregulation of translation and abnormal neuronal signaling, which associates with the cognitive and behavioral impairments observed in FXS [4]. Clinical phenotypes of FXS include facial dysmorphology, consisting of an elongated face, broad forehead, and large and prominent ears, and neurobehavioral impairment such as shyness, social avoidance and anxiety, attention deficit, hyperactivity, irritability, and aggression [5].

The methylation profile of the *FMR1* gene in individuals with FXS can vary even across cell types [6], and the completion of methylation appears to correlate with clinical phenotypes, especially with autistic features [7,8,9]. Incomplete silencing and mosaicism of FM, premutation (PM), or normal alleles are seen in most FXS cases [8,10,11,12,13].

Depending on the CGG repeat number and the methylation status, FXS individuals who have incomplete methylation and/or size mosaicism can present with lower FMRP and elevated *FMR1* mRNA, experiencing a double-hit mechanism, which correlates with cognitive impairments, psychiatric symptoms, and with co-occurring ASD [7]. In short, the behavioral phenotype of FXS exhibits a wide spectrum, in which fragile X molecular measures at the *FMR1* locus—such as CGG repeat size, methylation mosaicism, activation ratio (in females), and *FMR1* mRNA and FMRP expression levels—are known to be contributing factors for the variability of FXS phenotypes. However, environmental factors from pregnancy (toxins such as alcohol exposure) to developmental age (home environment, maternal/paternal psychopathology, educational/therapeutic services) can also contribute to the variability and heterogeneity of the behavioral phenotype [14,15].

Fetal alcohol spectrum disorder (FASD) is an umbrella term defining the range of adverse effects resulting from prenatal alcohol exposure. There are various diagnostic criteria for fetal alcohol spectrum disorders (FASDs), such as the University of Washington 4-digit Code, the 2016 Updated Clinical Guideline for FASD Diagnosis, the Diagnostic and Statistical Manual of Mental Disorders, Fifth Edition (DSM-5), and Canadian 2015 diagnostic systems [16,17,18,19]. FASD is classified into different subtypes based on the features identified and the diagnostic system used. Some of the diagnostic categories include fetal alcohol syndrome (FAS), partial fetal alcohol syndrome (PFAS), alcohol-related neurodevelopmental disorder (ARND), alcohol-related birth defects (ARBD), and neurobehavioral disorders associated with prenatal alcohol exposure (ND-PAE). The diagnosis of FASD involves a history of prenatal alcohol exposure and findings of facial abnormalities (i.e., short palpebral fissures, thin upper lips, and smooth philtrum), growth restriction, brain growth deficiency, and neurobehavioral impairments [20]. Children with FASD often struggle with neurocognitive issues (such as executive function deficits, and learning and memory problems), self-regulation challenges, and impairments in adaptive skills. FASD is often considered a hidden disability, as many children have average cognitive abilities and verbal skills, and only 10% exhibit the classic facial features described earlier [21]. In addition to the low prevalence of distinctive features that often lead to underdiagnosis, FASD shares several behavioral similarities with other neurodevelopmental disorders, including ASD [22,23], with approximately 72% of children with FASD meeting ASD criteria [24]. Epigenetic reprogramming and alteration of the glutamatergic pathway plays an important role in both FASD and FXS [25,26,27], resulting in facial dysmorphism and neurobehavioral impairments, which share a set of symptoms of behavioral deficits such as attention deficit, hyperactivity, irritability, and impulsivity in both FASD and FXS [25,26,27,28,29,30]. The estimated global prevalence of FASD is approximately 8 in 1000 individuals, and 1 in every 13 women who consumed alcohol during pregnancy will give birth to a child with FASD [31]. The prevalence of FASD in the U.S. population ranges from 11 to 50 per 1000 children [32]. Although there is no direct evidence estimating the prevalence of alcohol consumption among premutation carriers, some studies have reported that excessive alcohol consumption is relatively common in these individuals [33,34]. Therefore, children born to mothers with the premutation could be at high risk of being affected by both FXS and FASD. Additionally, prenatal alcohol exposure may have an additive effect, potentially affecting the severity of the clinical presentation in individuals with FXS.

Here, we present five siblings, two brothers with FXS and FASD and a triplet, two twins with FXS and FASD, and one fraternal triplet with FASD and no FXS. The mother was a carrier of an *FMR1* premutation allele and had a history of substance and alcohol use disorder. In this study, we compared the neurobehavioral characteristics of the five cases, and discuss the underpinning molecular pathways and the possible neurochemical alteration observed between FXS and FASD.

## 2. Results

**Figure 1:**
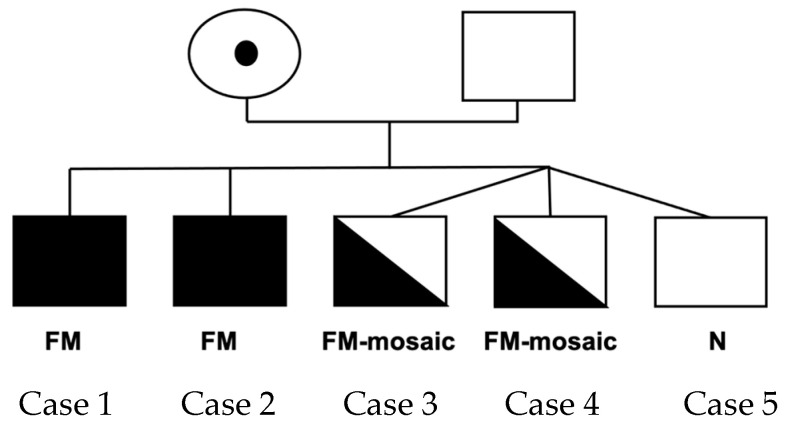
Pedigree including the five participants. FM = full mutation; FM—mosaic= full mutation mosaic; N = normal.

The five male adopted siblings were the children of a mother with the *FMR1* premutation (Pedigree Figure 1).

All children experienced multiple foster homes before being adopted by the current family. Thus, details of the prenatal and postnatal history of all cases are limited. In addition to the history of alcohol and substance (cocaine and opioid) usage by the biological mother during pregnancy, all cases experienced neglect, especially during the developmental age, that could have contributed to exacerbate their behavioral conditions. The cognitive, neuropsychiatric, and neurobehavioral evaluations of all cases are shown in Table 1.

**Figure 2:**
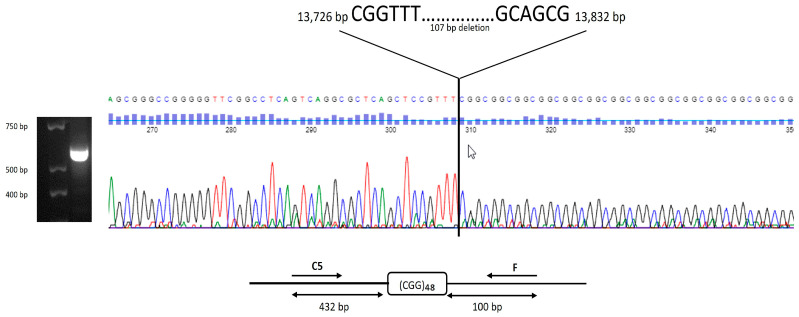
Graphical representation of the DNA sequencing results. Zoomed in electropherogram shows the sequence of the *FMR1* PCR amplicon (of 532 bp obtained using primer C5 and f) containing a de-letion of 107 base pairs upstream of the CGG repeat, observed in both the twin brothers with FXS (cases 3 and 4). The sequence in base pairs is displayed on the top of the image and different colored peaks show data for different bases (red for thymine, blue for cytosine, green for adenine and black for guanine). The presence of a deleted allele was demonstrated by PCR and visualized by gel electrophoresis (on the left of the figure) using primers C5 and f (also used for sequencing analysis). On the top, the exact locations, in bp, of deletion within the *FMR1* gene (L29074.1) is specified. The deleted allele retained the trinucleotide repeat of 48 CGG.

**Figure 3:**
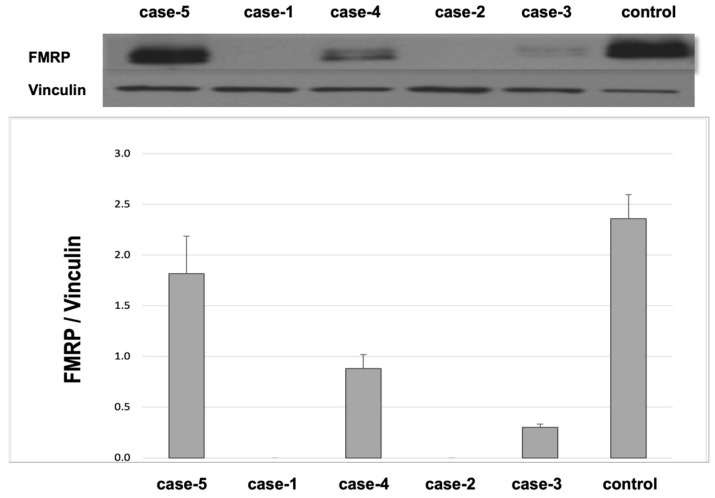
Western Blot analysis of fragile X messenger ribonucleoprotein of five cases. Western blot of FMRP and of loading control vinculin are shown on top. Histogram of FMRP levels is shown on bottom. Lane/bar 1 is case 5 (fraternal triplet, FASD), who shows normal FMRP level; lane/bar 2 is case 1 (FXS), with absent FMRP expression; lane/bar 3 is case 4 (triplet, mosaic FXS), with lower FMRP expression level; lane/bar 4 is case 2 (FXS), with absence of FMRP expression; and lane/bar 5 is case 3 (triplet, mosaic FXS) with low FMRP expression level, and lane/bar 5 is a 60 years old male control with 30-CGG repeat allele who shows normal FMRP expression level.

DNA testing showed that case 1 and case 2 had fully methylated FM alleles of >200 CGG repeats, with a consequent absence of FMRP. The two identical-twin triplets (cases 3 and 4) were FM—size mosaics, showing the presence of an FM (>200 CGG repeats) and the deletion (Figure 2) of a 107 bp genomic region upstream of a 48-CGG-repeat tract; methylation was observed in 70% and 60% of the cells, respectively, resulting in a low FMRP expression level (Figure 3). The fraternal triplet (case 5) showed a normal allele with 30 CGG repeats and an FMRP expression level in the normal range; see Figure 3.

### Cases

Case 1 is an 11-year-old child with a fully methylated FXS full mutation presenting with FXS, ASD, and FASD; specifically, he met the diagnostic criteria for alcohol-related neurodevelopmental disorder (ARND). Birth and early childhood information are unknown because he was taken away from his biological mother, who had history of drug and alcohol use disorder. His current behavioral phenotype includes self-harming, aggression towards other family members, low compliance, hyperactivity, and tantrums. He has a flat affect and appears depressed and anxious with reduced eye contact; he has a history of psychiatric hospitalization at 7 years of age, although suicidal behavior has not been noted. He is not yet toilet trained but has awareness of bladder and bowel movements. He has difficulties with sleep and displays socially inappropriate disinhibition. Physical examination shows FXS features including prominent ears, a mildly high palate, and flat feet with pronation. He does not have a long and narrow face or hyper-extensible joints. He has a history of growth restriction for weight and height (<10th percentile for his age). His current medications include guanfacine ER 4 mg, risperidone 4 mg, quetiapine 25 mg, and Adderall ER (amphetamine/dextroamphetamine) 30 mg.

Case 2 is a 9-year-old child with a fully methylated FXS full mutation presenting with FXS, ASD, and FASD; specifically, he met the diagnostic criteria for ARND, with significant self-injurious behavior. His medical history includes the surgical correction of gastroschisis at birth. Current challenging behaviors include head-banging and daily meltdowns. He has no history of suicidal behavior or seizures. He is also aggressive towards others and has trouble sleeping. He has poor eye contact and displays severe anxiety and irritability. Physical features include prominent and cupped ears, mishappened ear pinnae, epicanthal folds, right exotropia, hyper-extensible finger joints, a single palmar crease on the right hand, and flat feet. Physical measures are normal, with a remarkable low BMI of 15.6, and with growth restriction for weight and height (<10th percentile for his age) with poor weight and height gain. He is currently in a mainstream education class and receives a special pull-out class for math and English. Current daily medications include quetiapine 25 mg, guanfacine ER 4 mg, aripiprazole 7.5 mg am/10 mg pm, and risperidone 4 mg. He was tried on sertraline in the past but he became more aggressive, so sertraline was discontinued.

Case 3 is triplet 1, a 6-year-old child, full mutation-size mosaic, with *FMR1* alleles with >200 CGG repeats (60% methylation) and a deleted allele with 48 CGG repeats, but with 107 bp deletion upstream of the repeat present, presenting with FXS, ASD, and FASD, and meeting the diagnostic criteria for ARND. He was premature, with a low birth weight (3 pounds, 6 ounces). He had recurrent ear infections during early childhood, with no other known medical conditions. His current behavioral phenotype is notable for its aggression and he has limited language skills. He also has sleep disturbance, hyperactivity and problems with attention (ADHD), and severe anxiety. He is not yet toilet trained. Physical features include a broad and wide palate, slight clinodactyly of the fifth finger of his right hand, and hyper-extensible finger joints. Growth restriction is found for height and weight (<3rd percentile for his age). His current medications include clonidine, sertraline, trazadone, risperidone, and methylphenidate.

Case 4 is triplet 2, a 6-year-old child, full mutation-size mosaic, with *FMR1* alleles with >200 CGG repeats (70% methylation) and a deleted allele with 48 CGG repeats but with 107 bp deletion upstream of the repeat, presenting with FXS, ASD, and FASD, and meeting the diagnostic criteria for ARND. His birth weight was 3 pounds, 15 ounces. His medical history includes the surgical repair of an inguinal hernia at 3 years of age. Currently, his main behavioral difficulties include self-harming and aggression toward others, developmental delay, and delayed communication skills. He has problems with sleep and hyperactivity and attention (ADHD), and separation anxiety is also a concern for caregivers. He has repetitive speech and other stereotypic behavior. He is partially toilet trained. His physical exam was normal: he has a low height and weight (<3rd percentile for his age). His current medications include clonidine, trazadone, risperidone, and methylphenidate.

Case 5 is triplet 3, a 6-year-old with a normal *FMR1* allele with 30 CGG repeats, who presents with ASD and FASD, and meets the diagnostic criteria for partial fetal alcohol syndrome (PFAS). He has a history of language delay and is described as a picky eater. He had an extremely low birth weight (~2 pounds). He is currently of a small build, showing restrictions in growth (<3rd percentile for height and weight for his age) and not yet toilet trained. His current challenges include developmental delay, deficits in social communication, sleep disturbance, aggression, hyperactivity and attention (ADHD), anxiety, and recurrent tantrums. He has repetitive speech and poor eye contact. He has good learning skills. Physical exam reveals a flat and broad philtrum with thin lips, absent epicanthal folds, a broad palate, and slight clinodactyly in the fifth finger bilaterally. His current medications include clonidine, trazodone, and guanfacine.

## 3. Discussion

An expansion of the *FMR1* CGG repeat to greater than 200, called the FM allele, leads to *FMR1* methylation, gene silences, and the absence of gene transcription and translation and, thus, a lack of FMRP production. FMRP is a critical protein for typical brain function, functioning as a translational repressor at the synapses by inhibiting elongating ribosomes [35,36,37,38]. Its absence leads to increased protein translation in the brain, which leads to the FXS phenotype [39,40]. In this study, we report on the clinical and molecular features of five siblings. The older brothers (cases 1 and 2) have FXS with fully methylated FM alleles and no FMRP production, while the two identical-twin triplets (cases 3 and 4) are FM-size mosaics with low FMRP expression levels. The fraternal triplet has a normal *FMR1* allele and normal FMRP expression levels. All participants with FXS (cases 1, 2, 3, and 4) and with FASD (case 5) have ASD, with higher total ADOS scores (≥7) in all siblings who had FXS (cases 1, 2, 3, and 4) compared to case 5 (ADOS score 4).

The beginning of pregnancy is particularly vulnerable to environmental factor toxicity, which can lead to long-term effects for development [41]. Several factors can contribute to the complex phenotype of FASD, and the toxic effect of alcohol can be caused by genetic factors for susceptibility or resistance, which could ultimately affect the alcohol-induced phenotype, which means that mothers with a certain genotype could consume more alcohol [42]. In this study, all siblings were removed from their biological mother in early childhood and were in multiple foster homes and abusive situations that impacted their behavior before they were adopted together. The fact that case 5 did not have FXS but still had severe behavioral problems is related to both his FASD and his severe environmental exposures—including abuse in his biological and foster homes, in addition to his constant exposure to his severely affected siblings. He often copied his brother’s behaviors and yet he had the highest IQ because of his normal FMRP expression level. Since FASD can worsen in subsequent siblings as the mother ages because of ongoing damage to her liver leading to higher alcohol levels in blood, the triplets are likely the most affected by FASD; however, their higher FMRP levels could protect their IQ, most remarkably seen in case 5.

There is a significant variability in the behavioral phenotype and autistic features of individuals who have a fully methylated FM compared to those with FM mosaicism who carry unmethylated and methylated FM alleles, where some cells have a methylated allele that do not express FMRP and *FMR1* mRNA, and others carry unmethylated alleles which express FMRP and *FMR1* mRNA [8]. Further, elevated levels of *FMR1* mRNA lead to RNA gaining a toxic function, which may contribute to an increased risk of neurodevelopmental problems, including autism [28]. Cases 1 and 2 carry a fully methylated FM allele, and both have a high ADOS score, low non-verbal IQ, severe behavioral problems—especially irritability and hyperactivity –and a low adaptive behavioral score, in addition to externalizing behavior, i.e., aggression towards others, and tantrums. These severe clinical and behavioral problems are caused by accelerated conditions: in utero alcohol exposure during pregnancy (their brother, case 5, has FASD) combined with unfavorable environmental conditions (foster home environments, caregiver psychopathology, lack of stimulation/neglect), which may lead to modifying gene effects and potentially to epigenetic changes [43,44].

FMRP is an RNA-binding protein encoded by the *FMR1* gene that controls specific neuronal mRNA localization and translation, which is essential for proper synaptic plasticity and architecture [45]. The lack or absence of FMRP can cause the imbalanced release of glutamate and GABA from astrocyte cells, which may be involved in the neuropathology and underlying symptoms of FXS, such as cognitive impairment and the ASD phenotype [46,47,48,49].

Although several studies have reported on the presence of a small deletion in the genomic region spanning the CGG repeat in subjects with FXS [10,50,51,52,53,54,55,56], only a few have provided data on FMR1 mRNA and FMRP expression levels [56,57]. In our study, case 3 and 4 carry a deleted allele which includes a 107 bp genomic region upstream of the CGG repeats in approximately 30 and 40% of the cells, allowing the expression of 50% FMRP and 16% FMRP in case 3 and case 4, respectively. The small deletion upstream of the region of the CGG repeats did not cause gene silencing, and preserved both the transcription start sites [58] and the translation start site, thus producing FMRP in the cells carrying the deleted allele in both triplet twins [52]. Although the deletion of the entire CGG repeat region does not abolish expression [52], if both the transcription and translation start sites are unaffected by the deletion, the role of these deletions relative to FMR1 gene activity (transcription and translation) is not completely clear and requires additional investigations.

FMRP is essential for synaptic plasticity, which is critical for brain function [37]. Some studies have been concerned to discover the relationship between protein levels and the phenotypic characteristics of FXS, and lower FMRP levels have been documented in samples of individuals with FXS and ASD compared to patients with FXS only [59,60].

Glutamate, the most important excitatory neuron which plays an important role in synaptic plasticity and brain function, is the most affected neurotransmission system in ethanol-induced developmental disabilities, which work through a dual mechanism: the blockade of NMDA glutamate receptors and excessive activation of GABAA receptors, where ethanol has both NMDA antagonist and GABA mimetic properties [61]. The lack of FMRP in FXS individuals activates excitatory neurons, specifically metabotropic glutamate receptors (mGluR); thus, combined with excessive exposure to alcohol, it may cause individuals who carry a fully methylated FM allele to have severe behavioral concerns in addition to the classic FXS phenotypes, as seen in the cases reported here.

Alcohol use disorder is more common in males who carry the premutation (55–200 CGG repeats) as compared to the general population, possibly due to attempts to self-treat neuropsychiatric conditions like depression and anxiety [34]. Similarly, individuals with FXS have relatively high rates of anxiety and may be at higher risk for alcohol use disorder, especially in places where alcohol is consumed socially and limited psychiatric medications are available. One study reported on 20 men with FXS in Ricaurte, Columbia, 8 of whom showed significant alcohol consumption and 6 of whom met criteria for alcohol dependence syndrome [62]. In this study, exacerbating behavioral problems, including aggression and impulsivity leading to self-injury, was reported in the cases who met criteria for alcohol dependence syndrome. FXS is highly associated with intellectual disability and impulsivity, and these factors may also predispose individuals to alcohol dependence. Premutation carriers have an increased likelihood of alcohol consumption [63], which may increase the risk of FASD occurrence in children of female carriers, although further research in this area is needed. In this study, the biological mother is a premutation carrier who used alcohol and other substances during pregnancy. Female premutation carriers can experience the early onset of fragile X-associated neuropsychiatric conditions (FXAND), as compared to males, which may lead to self-medicating with excessive alcohol consumption [33].

The five siblings consist of two older siblings (cases 1 and 2) and the triplets, including two identical triplets (cases 3 and 4) and one fraternal triplet (case 5). All five siblings were diagnosed with FASD and were found to have significant growth restriction for both weight and height, which is not common in children with FXS. Thus, this is likely related to excessive alcohol exposure. In addition to having ASD, the two identical-twin triplets (cases 3 and 4) also had FXS and a higher ADOS score compared to the fraternal triplet (case 5), who also had the highest score for nonverbal IQ. The aberrant behavior and adaptive behavior were similar between triplets.

## 4. Materials and Methods

### 4.1. Subjects

All participants of this study were seen at the Fragile X Research and Treatment Center at the Medical Investigation of Neurodevelopmental Disorders (MIND) Institute, UC Davis Health Center, Sacramento, CA, USA. Legal guardians signed a consent form for the publication of this manuscript with their clinical details, evaluation findings, and photograph. Thorough evaluation included a clinical history and physical examination, assessed by an experienced developmental-behavioral pediatrician, and standardized neuropsychological testing by a qualified psychologist; parents completed standardized questionnaires.

### 4.2. Molecular Measures

Molecular analysis included DNA testing for the *FMR1* mutations by Southern Blot and PCR carried out on genomic DNA isolated from whole blood samples [64,65]. The fragile X messenger ribonucleoprotein (FMRP) expression level was measured using Western blot analysis in peripheral blood mononuclear cells (PBMCs) derived from participants as described in Zafarullah et al. [66]. Sequencing analysis was conducted by Sanger sequencing on gel-purified PCR amplicons obtained by PCR using primers C5 and F, as depicted in Figure 2. The primers’ sequence was as follows: C5: 5′ ATT TCC CAC GCC ACT GAG TG 3′; F: 5′ AGC CCC GCA CTT CCA CCA CCA GCT CCT CCA 3′.

### 4.3. Clinical Measures

The Leiter-3 test was administered to assess non-verbal cognitive functions [67]. The Autism Diagnostic Observation Schedule (ADOS-2) module was used to diagnose and classify the ASD [68]. FASD diagnosis was based on the 2016 Updated Clinical Guidelines for FASD Diagnosis, which includes four diagnostic categories, including FAS, PFAS, ARND, and ARBD. The Vineland Adaptive Behavior Scales, Second Edition (VABS-2) was administered to diagnose intellectual and developmental disabilities, while the severity of behaviors’ problems were rated using Aberrant Behavior Checklist-Community (ABC-C), a 58-item questionnaire for caregivers used to assess maladaptive behaviors [69,70]. Clinical details of the triplets, cases 3, 4, and 5, have been reported previously [63].

## 5. Conclusions

The phenotypic variability in the intellectual and behavioral phenotypes observed in the five siblings included in this study derived from a combination of the variability of the *FMR1* mutation characterized in the participants and environmental factors such as in utero alcohol exposure, which has likely played a role in worsening the behavioral phenotype.

All of the boys have severe behavioral problems compared to typical children with FXS, and these problems are likely exacerbated by their FASD, in addition to abuse from multiple foster homes.

Future direction: This study shows the impact of environmental factors, especially in utero and in early life, on behavioral phenotypes in individuals with FXS. Both FASD and FXS are risk factors of behavioral problems and ASD; therefore, having both may cause a double hit in the development of behavioral problems. In addition, previous reports demonstrate an increased rate of excessive alcohol use in premutation individuals, perhaps as a treatment for fragile X-associated neuropsychiatric conditions such as depression or anxiety. Accordingly, the education of family members, especially of carriers of a fragile X mutation, regarding background gene effects and lifestyle recommendations should be carried out, so that favorable lifestyle decisions can be made in adult life, in addition to avoiding excessive alcohol intake.

## Figures and Tables

**Table 1:** Summary of neuropsychological evaluations of five study participants.

Measurement	Case 1	Case 2	Case 3	Case 4	Case 5
Leiter-3					
Non-Verbal (NV)-IQ	69	57	61	55	84
ADOS-2 ^a^					
Social Affect (SA) Total	19	15	13	9	3
Restricted and Repetitive Behavior (RRB) Total	1	0	7	7	5
SA + RRB Total	20	15	20	15	8
Comparison Score	8	7	8	7	4
Classification	ASD	ASD	ASD	ASD	ASD
ABC-C ^b^					
Irritability	40	40	38	39	36
Lethargy/Social Withdrawal	22	10	4	5	3
Stereotypy	10	10	10	10	12
Hyperactivity	31	41	37	43	37
Inappropriate Speech	5	6	4	7	6
Composite Score	108	107	93	104	94
Vineland					
Communication Standard Score	28	50	62	60	62
Daily Living Skills Standard Score	59	67	72	71	64
Socialization Standard Score	38	50	48	50	48
Adaptive Behaviour Composite	43	57	62	61	59

^a^ Autism Diagnostic Observation Schedule. ^b^ Aberrant Behaviour Checklist—Community.

## Data Availability

Data are contained within the article.
